# The Role of Oxytocin in Domestic Animal’s Maternal Care: Parturition, Bonding, and Lactation

**DOI:** 10.3390/ani13071207

**Published:** 2023-03-30

**Authors:** Daniel Mota-Rojas, Míriam Marcet-Rius, Adriana Domínguez-Oliva, Julio Martínez-Burnes, Karina Lezama-García, Ismael Hernández-Ávalos, Daniela Rodríguez-González, Cécile Bienboire-Frosini

**Affiliations:** 1 Neurophysiology, Behavior and Animal Welfare Assessment, DPAA, Xochimilco Campus, Universidad Autónoma Metropolitana, Mexico City 04960, Mexico; 2 Department of Animal Behaviour and Welfare, Research Institute in Semiochemistry and Applied Ethology (IRSEA), 84400 Apt, France; 3 Facultad de Medicina Veterinaria y Zootecnia, Universidad Autónoma de Tamaulipas, Victoria City 87000, Mexico; 4 Facultad de Estudios Superiores Cuautitlán, Universidad Nacional Autónoma de Mexico (UNAM), Cuautitlán 54714, Mexico; 5 Department of Molecular Biology and Chemical Communication, Research Institute in Semiochemistry and Applied Ethology (IRSEA), 84400 Apt, France

**Keywords:** maternal care, maternal aggression, mother–young bonding, milk ejection

## Abstract

**Simple Summary:**

Oxytocin is one of the most important hormones in birth and lactation. Its importance lies in the fact that it is also involved in various bodily functions around parturition and establishing maternal behavior. We aimed to analyze all the facts involving oxytocin regulation and participation in different peripartum situations in domestic mammals. In addition, the authors examined the impact of altering normal plasma oxytocin values on maternal behavior and care, productive parameters, and imprinting.

**Abstract:**

Oxytocin (OXT) is one of the essential hormones in the birth process; however, estradiol, prolactin, cortisol, relaxin, connexin, and prostaglandin are also present. In addition to parturition, the functions in which OXT is also involved in mammals include the induction of maternal behavior, including imprinting and maternal care, social cognition, and affiliative behavior, which can affect allo-parental care. The present article aimed to analyze the role of OXT and the neurophysiologic regulation of this hormone during parturition, how it can promote or impair maternal behavior and bonding, and its importance in lactation in domestic animals.

## 1. Introduction

Oxytocin (OXT) is a uterotonic hormone in the endocrine response during parturition [1]. It is synthesized in the hypothalamus, in the paraventricular (mainly from its magnocellular cells) and the supraoptic nucleus [2], and transported through the neuro-hypophysial bundle to the posterior lobe of the pituitary gland, where it is stored in secretory vesicles before being released into the peripheral circulation [3]. Then, it induces myometrium contractions due to its action on myoepithelial cells [4,5]. It regulates, together with other hormones (e.g., estrogen and progesterone (P4)), the reproductive function of domestic animals [6]. In addition, there is a central action of OXT through dendritic and/or axonal releases within the brain. Smaller parvocellular neurons in the paraventricular nucleus also produce OXT and project directly to other regions in the brain, such as the limbic system (notably the amygdala and hippocampus) and the nucleus accumbens [3]. The dual action of this nonapeptide as a peripheral hormone and a central neuromodulator/neurotransmitter [7], as well as the wide distribution of OXT receptors (OXTR) in various brain areas and peripheral organs, indicate that OXT is involved in diverse biological functions and, notably, OXT influences the parturition process, the immediate establishment of maternal recognition, and milk ejection [8,9,10].

In mammals, several species have been evaluated to study the association between OXT concentration and maternal care [11], social cognition, and affiliative behavior [12,13], including nursing non-filial offspring or allo-parental care [14,15]. According to some authors, the positive relationship with close social partners is associated with alterations in peripheral OXT in dogs (*Canis familiaris*) [16] and chimpanzees (*Pan troglodytes*) [17,18].

The degree of interaction between parturition and maternal attachment to OXT concentrations is suggested to be related to a high density of oxytocinergic neurons in the brain, which would have a beneficial effect on the behavior of the mother in facilitating maternal recognition, protection of the offspring, and even habituation to the environment [19,20,21]. However, it is unclear whether this behavior is only induced by the action of OXT or is coordinated by the effect of hormonal peaks present before parturition that could sensitize OXTR. In the same way, OXT actively participates in lactation because it facilitates milk ejection and can even help induce the care of offspring from other mothers [22,23].

The present article aimed to analyze the role of OXT and the neurophysiologic regulation of this hormone during parturition, how it can promote or impair maternal behavior and bonding, and its importance in lactation on domestic animals, including pets and livestock.

## 2. Neurophysiological Regulation of OXT

**Figure 1:**
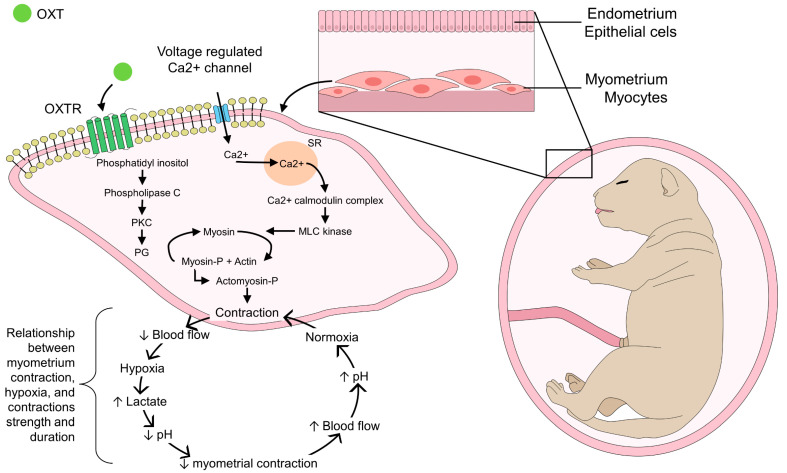
Mechanism of action of oxytocin during myometrial contractions. During parturition, the activation of OXTR at the uterus by OXT starts myometrial contractions. The interaction between OXTR and voltage-regulated Ca^2+^ channels enables the formation of the actomyosin-P complex to produce myocytes’ contraction. After contraction starts, a relation between contraction maintenance and uterine blood flow is responsible for maintaining effective contraction movements and, therefore, a normal course during parturition. In the first instance, myometrial contractions cause compression of uterine blood vessels, reducing blood flow. Hypoxia induces an acidemia state, by lactate increase, which decreases Ca^2+^ influx and, consequently, myometrial contractions stop, restoring blood flow and normoxia, in order to start another effective contraction. The ongoing maternal and fetal stimulation that promotes OXT release maintains rhythmic contractions due to initial hypoxia and acidemia of the myocytes, an effect that decreases contractions, and the cycle begins again when reaching normoxia and a regular uterine blood flow. MLC: myosin-light chain kinase; OXTR: oxytocin receptor; PG: prostaglandins; PKC: protein kinase C; SR: sarcoplasmic reticulum.

OXT is a cyclic neuropeptide of nine amino acids whose chemical structure contains a cyclic disulfide essential for its biological effect [24]. It has a structural similarity to vasopressin (AVP), with a minimum difference of two amino acids, which explains why they share physiological effects [25]. One of the main functions of OXT as a neurotransmitter and neuromodulator is during parturition, lactation, and maternal behavior, due to its uterotonic effect [22,26,27,28] (Figure 1).

From the paraventricular nucleus (PVN), where OXT is synthesized, OXT is transported to the neurohypophysis as a precursor molecule in oxytocin-neurophysin. It is later catalyzed to its active form with the activity of the enzyme monooxygenase peptidyl glycine α-amidating [29].

OXT synthesis does not only occur at the hypothalamic level, since its synthesis has also been recorded, to a lesser extent, in the corpus luteum, ovary [30], and testicles [31]. Other elements, such as estrogens, are also involved in regulating OXT. For example, estrogens downregulated OXT gene expression in the PVN and supraoptic nucleus (SON) [32,33]. This is due to the presence of estrogen receptors (ER) in oxytocinergic neurons of the PVN. In mice, ERbeta is necessary to regulate the expression of OXT and arginine vasopressin (AVP) [34]. This is relevant considering that both hormones participate in social interactions, fear reactions, and stress-related behaviors and depend on their upregulation to activate OXT and AVP systems [35,36]. Neuropeptide synthesis also takes place in cardiovascular tissues, as reported by Jankowski et al. [37] when studying OXT concentrations in the pulmonary artery and vena cava in male rats and the aorta of dogs and sheep. The results indicated concentrations of 2745 ± 180 pg/mg in all tissues studied, confirming their presence by PCR.

Additionally, high levels of OXT mRNA receptors were observed in the vena cava, vein, and pulmonary artery, indicating specific sites of action. These findings suggest the role of OXT in regulating vasomotor tone due to its natriuretic properties. In the heart, Jankowski et al. [38] studied cultures of rat cardiomyocytes with a concentration of OXT 19 times higher than in utero. In an immunostaining test on atrial myocytes and fibroblasts, the intensity was found to parallel atrial natriuretic peptide stores. Its presence in this tissue supports its vasomotor effect and possible regulation of cardiovascular activity [38].

OXT secretion also occurs at the brain level in the dendrites of magnocellular neurons. These neurons express a more significant amount of protein products of the immediate early c-fos gene. This effect was reported by Douglas et al. [39], who used forced swimming in 16- or 17-day-old virgin female rats as a stressor to investigate the influence of the event on OXT levels. The authors found that this factor increased OXT at 5 min post-test. These results suggest that this neuropeptide activates the hypothalamic–pituitary–adrenal (HPA) axis during parturition and potentially stressful events [40].

Concerning this, Ochedalski et al. [41] determined the effect of OXT in 7-day-old ovariectomized rats that received an infusion of intracerebroventricular OXT (100 ng/h), in addition to administration of estradiol (E2) with or without progesterone replacement. These authors found that E2 increased plasma adrenocorticotropic hormone (ACTH) and corticosterone concentrations. This was also associated with increased estrogen and increased corticotropin-releasing factor (CRF) mRNA in the PVN and mRNA of opiomelanocortin (POMC) in the pituitary gland. In another study, the administration of OXT reduced levels of plasmatic ACTH, CRF, and POMC in response to the elevation of E2 due to stress. Therefore, these findings suggest a modulating effect of OXT in response to a stressor due to actions on the HPA axis [42].

The above mentioned studies show the complex functions and interactions of OXT in the neurobiology of the organism. Brunton [28] points out that one of the mechanisms that restrict OXT secretion is regulated through endogenous opioids. These act centrally on the axonal terminals of the posterior pituitary, which has been reported in bovines with opioidergic receptors in neuronal terminals of the neurohypophysis, supraoptic neurons, and paraventricular in the hypothalamus [43]. The presence of these receptors in contiguous regions in oxytocinergic neurons has been demonstrated in other studies, such as that developed by Douglas et al. [44] in pregnant rats from 18 to 21 days. In these animals, a k-receptor agonist and an opioid antagonist, such as naloxone, were administered, reporting that agonism decreased OXT levels while antagonists increased its concentration. These same authors mention that, during the last week of gestation, the sensitivity towards these receptors decreases, probably as a mechanism to allow neurosecretion of OXT during delivery.

In addition, a study on the interaction between OXT and nitric oxide (NO) has shown that the increase in NO can lead to a decrease in c-Fos expression and, consequently, inhibit OXT secretion [45]. Srisawat et al. [46] evaluated the effect of an inhibitor of a NO-synthase in anesthetized virgin rats, identifying that the administration of this compound facilitated the secretion of OXT and that its use, together with nitroprusside (NO donor) in the supraoptic nucleus, inhibited the electrical activity of oxytocinergic neurons.

Therefore, since the chemical structure of OXT is similar to AVP, it is possible to understand its participation in cardiovascular control processes and stress-related events such as parturition, known as a eustress process [1,25]. Likewise, OXT modulation during stress perception indicates the participation of this hormone in the complex physiological process of parturition [8,10].

## 3. OXT Participation at Parturition

In all mammals, the perinatal period is characterized by various hormonal changes, including increases in plasma E2, prolactin (PRL), and cortisol, as well as activation of the oxytocinergic system at parturition [47,48]. This activation increases OXT during parturition in mammals [49], especially in dogs, where OXT plasma concentration is tightly implicated in uterine contractions. This can be useful to detect dystocic parturition opportunely [50]. Unlike in cattle, plasma cortisol levels in dogs are highly variable during the peripartum, higher than during prepartum luteolysis [51]. Olcese and Beesley [52] found that melatonin has a synergetic action and enhances oxytocin-induced contractions binding to specific melatonin receptors (MT2R) in the myometrium. This change involves physiological, neurological, morphological, hormonal, and behavioral levels [53].

There are important interrelationships between OXT and prostaglandin (PGs). OXT has been shown to stimulate PG release in many animals, primarily in the uterine epithelium/decidua. The effects of OXT are mediated by the presence of the tissue-specific OXT receptor, causing myometrium contraction and synthesis of PG in the decidua [49]. However, uterine contractions involve several hormones, including OXT and prostaglandin E2 (PGE2), connexin, progesterone (P4), and estrogen [54,55]. Connexin 43 (Cx43) is a gap junction protein distributed in the myocardium and uterus [56]. According to Chan et al. [57], OXT can stimulate the synthesis and release of PGE2 and PGF2α, increasing the susceptibility of the uterus to OXT and strengthening subsequent uterine contractions. However, it is widely recognized during labor that OXT is the primary hormone that promotes the synchronization of uterine contractions throughout labor and the subsequent dilation of the cervix [8,27,58], although the control of contractions in the myometrium is innervated by the hypogastric nerve, which provides sympathetic innervation that coordinates uterine contractions during the first phase of labor through agonism of alpha-adrenergic receptors [2].

The OXTR increase during pregnancy in the endometrium and myometrium has been reported in various species, e.g., in humans [59], rats [60], sheep [61], bovines [62,63], bitches [64], and pigs [65]. OXT is responsible for inducing the most violent and constant contractions, so that the fetus is expelled from the uterus to the outside [66].

In a study by Fuchs et al. [62], the endometrial OXT and AVP receptor concentrations were inversely correlated with plasma P4 concentrations (*p* = 0.005) with no correlation to plasma E2. In contrast, the myometrial receptor concentrations showed no correlation with plasma P4 but an inverse correlation with plasma E2 (*p* = 0.004). Similarly, in a study by Ou et al. [60], the authors found that mRNA expression of OXTR in the myometrium of rats during pregnancy and parturition is regulated by coordinated interactions between mechanical and endocrine signals. In another study by Meier et al. [61] in ewes, the authors reported that oxytocin-induced 13,14,dihydro-15-keto PGF2 alpha release during early gestation is minimal, despite the presence of endometrial OXT receptors. In mid-gestation, oxytocin-stimulated 13,14,dihydro-15-keto PGF2 alpha release is increased, with a concomitant increase in uterine OXT receptor concentrations [61].

The clearest example of the participation of different hormones during parturition is the relationship between estrogen and OXT. Masoudi et al. [67] evaluated the effect of E2 and OXT treatment on cervical dilation in three sheep breeds. In animals that were administered estrogens, no effect was observed on the dilation of the cervix. In contrast, animals supplied with estrogens and OXT allowed complete cervix dilation. What was shown by these authors is likely to be that the increase in estrogens would lead to higher concentrations of OXT. However, a study by Veiga et al. [64] evaluated the expression of estrogen mRNA and OXT mRNA genes in bitches during pregnancy or parturition. They observed that the mRNA expression for estrogens did not differ.

Nevertheless, mRNA expression for OXT increased during the last week of gestation. These results demonstrated that the estrogen peak during labor would not be related to the activity of OXT on the myometrium. Fuchs et al. [63] maintain that, at least in sheep and cattle, the increase in estrogens can increase the production of OXT in the magnocellular nuclei of the hypothalamus and facilitate the release of OXT from the posterior pituitary lobe. This might suggest that estrogen secretion may help coordinate the rate and release of OXT during labor.

**Figure 2:**
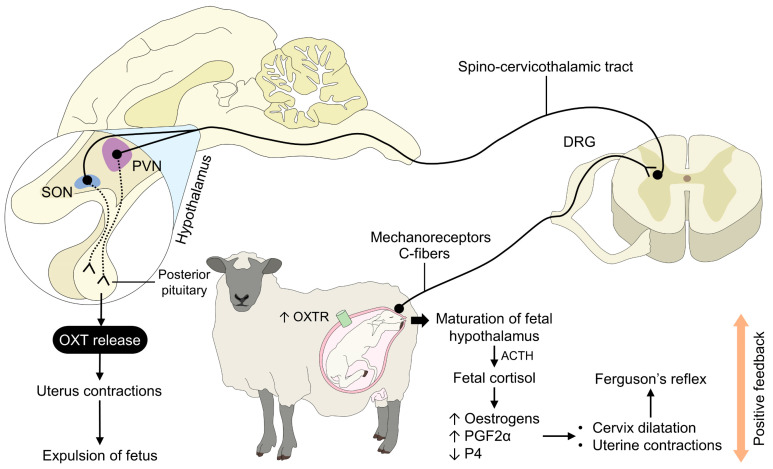
The role of oxytocin during parturition. In the final phase of pregnancy, secretion of ACTH due to the maturation of the hypothalamus leads to the release of fetal cortisol. These hormones also respond to the increase in E2 and PGF2α, and the decrease in P4. These hormones promote cervix dilation and the mechanoreceptors’ stimulation in the uterus. The sensory signals are processed in the PVN of the maternal hypothalamus after its transmission through fibers in the spinal cord. The interaction between the hypothalamus and the pituitary causes OXT release by the posterior pituitary into the bloodstream to initiate the physiological process of fetal expulsion. DRG: dorsal root ganglion; OXT: oxytocin; OXTR: oxytocin receptors; P4: progesterone; PGF2α: prostaglandin F2α; PVN: paraventricular nucleus; SON: supraoptic nucleus.

Some stimuli are involved in the release of OXT; the first is the Ferguson reflex, which consists of the pressure of a puppy’s head on the cervix during parturition [68], cervicovaginal stimulation during labor and PRL secretion, along with the presence of E2 and progesterone, releasing OXT by producing reactions in the maternal brain [69]. The second stimulus is breastfeeding stimulation of the mammary glands (MG) by the pups [68] (Figure 2).

It is also important to mention that levels of OXT could influence the interval of expulsion or the total parturition time, as reported in sows and bitches. In sows, low levels of OXT depend on environmental factors influencing the farrowing phase, such as being confined in crates (38.1 ± 24.6 pg/mL) rather than pens (77.6 ± 47.6 ng/mL), having a direct effect on farrowing duration (*p* < 0.001) [70]. This was also reported by Yun et al. [71] when providing nesting material to sows, promoting high non-esterified fatty acid concentrations correlated with OXT levels (r_s_ = 0.28) and piglet colostrum intake, potentially improving their immune system and survival. For example, in sows, low levels of OXT can increase the duration of farrowing. According to Alonso-Spilsbury et al. [72], OXT is at lower levels during prolonged farrowing, causing increased piglet mortality in intensive production systems [73]. Similarly, van Dijk et al. [74] observed that the mortality percentages increased statistically significantly in prolonged intervals of piglet expulsion, and with stillbirths or posteriorly presented fetuses, the mortality percentages also increased statistically significantly. This prolonged farrowing can cause higher stillbirth frequency, thereby increasing economic losses in intensive pig farming [2]. In the case of pregnant bitches, low levels of neuropeptide could cause uterine inertia, which is approximately 75%, the most common maternal cause of dystocia in the pregnant bitch. This problem could be due to the absence of myometrial contractions, some obstruction [75,76], or myometrial exhaustion caused by an obstruction after several pups have been expelled [77]. From all the above, it can be understood that, although various hormones are involved in the birth process, OXT is one of the most important in all the reviewed species.

## 4. The Role of OXT in the Establishment of Maternal Behavior Regulation

**Figure 3:**
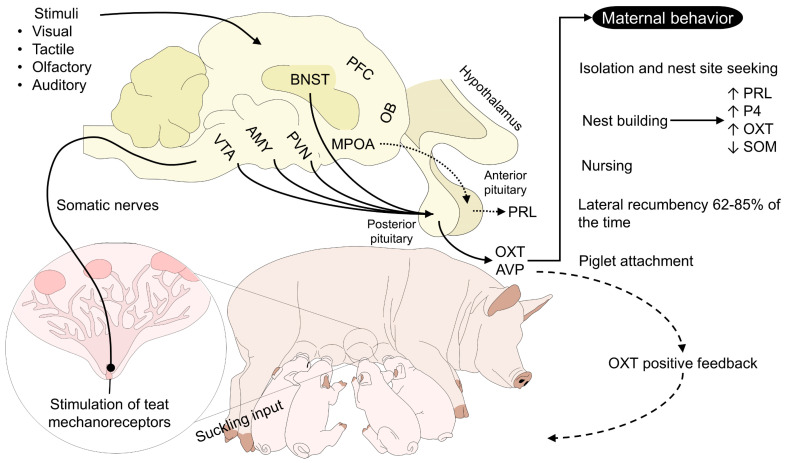
The oxytocinergic positive feedback loop of maternal behavior in sows. The visual, tactile, olfactory, and auditory stimuli that are constantly perceived by the sow participate in maternal responsiveness and attachment due to the activation of vital cerebral regions sensitive to OXT. For example, stimulation and activation of AMY, BNST, PVN, and VTA promote OXT release in the posterior pituitary. After OXT is released, several peripartum maternal behaviors in mammals are influenced by this hormone. In the case of sows, increases in circulating OXT levels before the onset of farrowing motivate nest site seeking and nesting. Particularly, nest building has also been associated with high concentrations of OXT, P4, PRL, and low levels of SOM in aforementioned cerebral regions. After farrowing, the development of maternal attachment and nursing of the piglets requires so-called OXT positive feedback, where piglets stimulate teat mechanoreceptors through suckling, releasing OXT in the maternal brain and promoting maternal behaviors. On the other hand, MPOA axons project to the anterior pituitary to release PRL and participate in milk secretion. AMY: amygdala; AVP: arginine vasopressin; BNST: bed nucleus of the stria terminalis; MPOA: medial preoptic area; OB: olfactory bulb; OXT: oxytocin; P4: progesterone; PFC: prefrontal cortex; PRL: prolactin; PVN: paraventricular nucleus; SOM: somatostatin.

Maternal care is a series of species-specific behavioral patterns to ensure the offspring’s survival [78]. They include interactions such as nursing, attention, and protection of the neonate [20]. The hormonal cascade and the activation of the MPOA can vary among mammalian species [48]; however, sex steroids synthesized by the ovaries, OXT, AVP, and PRL are present in the maternal brain. High levels of P4 and low E2, PRL, and OXT are present during gestation. Contrarily, in cows, P4 diminishes a couple of days after parturition, and E2, PRL, and OXT increase at the onset of parturition and the first post-partum days [79] (Figure 3).

OXT is involved in the different phases of parturition and is considered an essential hormone to facilitate maternal bonds [80]. In several studies, disrupted brain OXT signaling is linked to poor mothering [81]. OXT evaluation can be performed in domestic animals through plasma, saliva, and milk [82]. In sows, behaviors such as nest building, pawing, and gathering straw are used to appraise maternal aptitude [83]. Hall et al. [84] determined in Landrace x White Large sows that the mean concentration of OXT in milk was higher than in saliva samples (38.32 vs. 29.60 pg/mL). However, both sampling methods registered the highest concentrations during the first four to five post-farrowing days, a trait that can be associated with the specialized maternal care that newborn piglets require during the first days of life [85]. This finding was similar to a study reporting that salivary OXT concentration was significantly higher during the first day after farrowing (1654 pg/mL) than after nine days (1142 pg/mL) [86]. From an endocrine perspective, the first post-partum days are usually accompanied by higher OXT signaling due to the positive feedback loop initiated by suckling [87]. The positive OXT feedback from the newborns’ suckling also stimulates OXT release in several mammals [88,89]. Milking behavior predominates during the first seven days post-parturition, as reported in sows and ewes [90,91].

Although OXT is considered essential for maternal care, which includes nest-building, reluctance to leave the nest, genital and overall licking of the newborn, nursing, and direct contact with the litter [92] in mammalian species, the OXT action can pass from crucial to nonessential, depending on the neurodevelopmental stage at birth or the amount of help the dams can receive from other members of the herd [87,93], and can help not only with the establishment but also with the maintenance of the maternal behavior [94]. Ungulates build highly selective maternal care for their offspring. They do not usually receive or allow allomaternal care (known as the nursing of non-filial offspring [95]), although, recently, Orihuela et al. [96] reported that communal rearing was observed in zebu cattle. In this species, maternal bonding and OXT are suggested to have an essential role [97].

For example, licking, nursing, and low-pitched bleats are rapidly observed (during the first minutes post-parturition) in ewes and goats. These behaviors are part of the maternal responsiveness to the hormonal cascade started by vaginocervical stimulation, OXT secretion, and activation of cerebral structures such as the main olfactory system (MOB), MPOA, and PVN [98]. Likewise, vaginocervical stimulation in ewes increases OXT in limbic structures that participate in maternal receptivity. However, in non-gestant animals, intraventricular administration of OXT is indispensable to nursing and accepting suckling [99]. When considering levels of OXT in the cerebrospinal fluid (CSF) of ewes, these were 15% significantly higher than plasma levels during lambing, while plasma concentrations only increased within 15 min post-lambing [100]. Some authors have hypothesized that differences could be found even within species destined for different zootechnical purposes (e.g., beef or dairy cattle). Geburt et al. [80] assessed salivary OXT concentrations in 20 Simmental beef-type and 20 German Black Pied dairy-type cows to correlate its level to maternal behaviors, such as the intention to defend the calf. The results showed that dairy cattle had higher OXT concentrations (88.6 ± 9.2 vs. 62.8 ± 9.2 pg/mL) and higher dam–calf interactions (13.8 ± 1.4) but lower defensive behaviors towards the newborn. Additionally, the differences between both types of cattle were not statistically significant at three days post-calving, so the authors concluded that behavior and OXT concentration need further assessment to consider them objective biomarkers for maternal behavior.

Apart from OXT, PRL and somatostatin have also been studied as markers of maternal behavior in sows and ewes. In sows, blood OXT increases (*p* = 0.002), PRL decreases (*p* = 0.005), and no change in somatostatin (*p* = 0.65) were reported during nursing [101]. Furthermore, the relation between OXT and other hormones is not limited to maternal traits since the levels of OXT were correlated to the piglet’s weight gain, showing that this hormone influences the lactation process and piglet growth [101]. As for PRL and cortisol, environmental enrichment in sows before and after farrowing increased OXT and PRL concentrations (44.3 ± 5.67 ng/mL and 19.6 ± 3.61 ng/mL, respectively) while decreasing cortisol levels. These results were correlated to nest construction behavior, in which prepartum pigs in the enrichment group had higher frequency and periods of nesting (*p* < 0.01), as well as longer times in lateral recumbency after farrowing (a behavior that promotes suckling from the piglets) [102]. In ewes, along with OXT, arginine-AVP levels were evaluated, finding that plasma concentrations increased during lambing and 15 min post-lambing [100].

In contrast to livestock, in non-domestic species such as prairie voles, increased plasma OXT levels were obtained in reproductively naïve male individuals after 10 min of pup exposure, activating oxytocinergic neurons in the paraventricular nucleus (PVN) of the hypothalamus [103]. A high quantity of OXT receptors in the nucleus accumbens of virgin female prairie voles has also been associated with females showing spontaneous maternal behaviors such as lick and pup grooming (*p* < 0.05), indicating that OXT concentration has a secondary role in developing maternal traits in these species [104]. Its role during reproductive events has been reported in grey seals, where significantly lower concentrations of OXT (4.3 ±0.5 pg/mL) were found in non-breeding females than in those during early and late lactation (8.2 ± 0.8 pg/m and 6.9 ± 0.7 pg/mL, respectively) [105]. Therefore, as mentioned in a heuristic model by Taylor and Grieb [93], the importance of OXT to mothering is higher in sheep/goats, cows, rats, mice, prairie voles, and marmosets.

Since OXT is essential to establish positive interactions with newborns, alterations or decreasing levels of this hormone have been associated with maternal aggression and cases of cannibalism. In a study made in Kangal dogs, cannibalism has been associated with low concentrations of plasma OXT (3.58 ± 0.43 pg/mL), as well as cholesterol (125.50 ± 8.6 mg/dL), high-density lipoproteins (52.00 ± 9.34 mg/dL) and low-density lipoproteins in serum (30.45 ± 3.56 mg/dL) [106]. Negative traits in ewes, such as butting and moving them away from the lamb, were recorded in animals without vaginocervical stimuli that did not receive P4 or estrogen [107]. Contrarily, McLean, et al. [108] reported that sex steroids do not relate to aggressive bouts in parturient gilts. Conversely, in rodents, OXT knockout mice have shown reduced aggression, and administration of OXT increased the number of attacks on pups at the beginning of the lactation. At the same time, high AVP levels and blockade of AVP receptors reduced maternal aggression [109].

According to the species in question, the results can be variable regarding the function of the OXT. Although OXT and other biomarkers such as PRL, AVP, or cortisone are used to study maternal behavior, some limitations exist [78], as Ogi et al. [7] reported in 25 Labrador Retriever dogs. They found no association between salivary OXT levels and maternal behaviors, except for sniffing or poking the newborn. At the same time, other studies relate OXT to interest in the newborn and anxiety reduction [8]. In the present authors’ opinion, the conclusions of these studies can be applied to all species: maternal behavior (and aggression) is highly influenced by the simultaneous activation of the endocrine and sensory systems in triggering care for the newborn and developing the mother–newborn bonding.

Bonding is a process by which the dam and the offspring establish a social preference [110,111]. It has been initially studied in birds and later in sheep, buffalo, deer, and other non-domestic species such as insects [112]. In mammals, this preference constitutes mutual recognition to guarantee the young’s development through adequate maternal care [20,95]. Since the mother is the main caregiver in most species, a significant part of knowledge about the neurobiological and neuroendocrine control of bonding actions is based on the maternal response [113,114,115,116]. Through this bonding, the neonate perceives and acquires a multisensory image of its mother, building a selective and preferential behavior towards her [117,118].

Maternal recognition occurs in precocial and altricial animals; however, the level of autonomy at birth and their early learning ability determines the type of bond in domestic animals, wildlife, and companion animals [119,120,121]. Three processes are required to establish the female bond: an increased acceptance of the individual, a reduced fear or low rejection reaction, and a motivation to care for the neonate [79]. To accept the offspring immediately after parturition, sensorial stimuli, such as visual, olfactory, gustatory, and auditory cues, activate brain structures such as the locus coeruleus, some areas of the limbic system, olfactory bulb, auditory cortex, and visual cortex [22,122], and release neurotransmitters to modify the learning process [120,121,123].

OXT participates during dam–young bonding and simultaneously acts peripherally and centrally with other hormones from pregnancy to postpartum [124,125]. For this reason, OXT is recognized as the primary neurochemical substance associated with affiliative and learning behaviors [119]. Gamma-aminobutyric acid, glutamate, and acetylcholine are other neurotransmitters which have been studied during this period [111].

The so-called sensitive period, which can last a few hours or days depending on the species, is the interval in which both the female and the offspring are more receptive to selective recognition [123,126]. In ewes, this period implies that early dam-calf bonding depends on maternal and neonatal behaviors, social interaction, and neuroendocrine modifications, mainly led by OXT [122,127,128,129].

For mammals, olfactory recognition is one of the leading systems to establish the maternal bond, due to the interaction between the central olfactory system and OXT release by the anterior pituitary [130]. OXT and other nonapeptides, such as AVP, promote maternal care by acting in cerebral structures at the bed nucleus of the stria terminalis and medial amygdala [79], emphasizing that only the central olfactory system participates during odor discrimination of offspring [130]. This has been studied in ewes, in which the selective bond through olfactory cues occurs during the first two to four hours after lambing [131], and suppression of olfactory neurogenesis impairs maternal acceptance [132]. Keller et al. [133] reported that visual/auditory recognition is highly affected by the maternal experience. In contrast, olfactory stimuli occur indistinctly between 30 min to 4 h post-lambing in primiparous and multiparous females, and the release of OXT from the PVN triggers maternal attachment [98].

In this sense, Kojima et al. [134] observed in laboratory rats that OXT brain levels in the litter can be modified to establish recognition of their mother. At the same time, the contact translates into mutual recognition [135]. When the lambs do not have an adequate amount of fetal fluids, the ewes do not need to clean them, and this leads to a decrease in the release of P4, E2, and OXT, because there is not so much physical communication, which stimulates lactation [136]. Therefore, maternal attachment is a process in which OXT has an essential role. However, it also depends on the endocrine control of other elements, such as OXT (opioids, estrogens, among others) and multisensorial cues [137]. As stated by Lévy [48], maternal behavior in non-human and human animals is mediated by hormonal mechanisms, their interaction with crucial cerebral regions such as the MPOA, and the interaction with the newborn.

## 5. Role of OXT in Lactation

**Figure 4:**
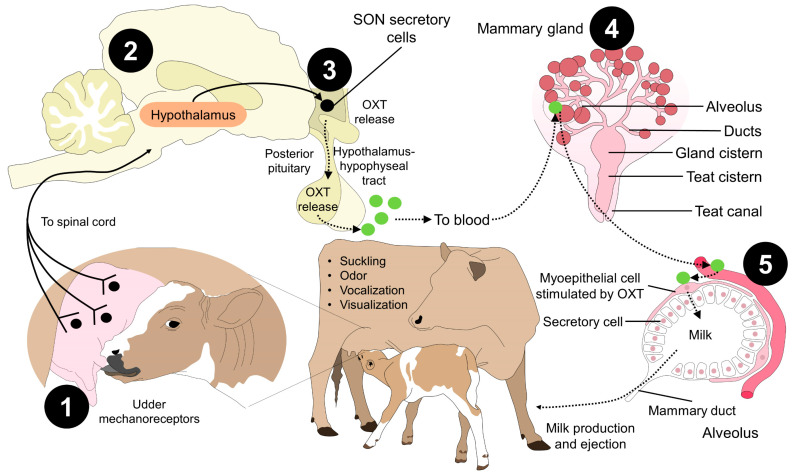
Neurophysiology of milk ejection and oxytocin influence. Milk yield and ejection can be divided into five stages. 1. Environmental stimulus, such as suckling from the calf, activates udder mechanoreceptors in the teat. 2. Through nervous tracts, the sensory signal reaches the hypothalamus. 3. The SON secretory cells are activated to release OXT. 4. When OXT reaches the bloodstream, it acts on the myoepithelial cells of the alveolus, causing the contraction and secretion of milk by the secretory cells (5). This physiological path and the aforementioned stimulus from the offspring are one of the reasons why manual or mechanical stimulation of the udder is important in dairy systems and can depend on OXT administration, regardless of its controversial effects. OXT: oxytocin; SON: supraoptic nucleus.

A neuro-hormonal reflex produces milk ejection: this comprises an ascending neuronal pathway from the nipples to the hypothalamus and a descending vascular link that transports pituitary hormones, especially OXT, to the mammary gland (MG) [138]. Milk ejection occurs after stimulating and activating pressure-sensitive receptors in the inguinal canal at a neurophysiological and neuroendocrine level. These have projections towards the dorsal roots of the spinal cord, which in response promote, via the release of OXT into the bloodstream from the posterior pituitary, the contraction of myoepithelial cells at the alveolar and ductal level, increasing intra-alveolar pressure and minimizing resistance to milk flow, descending to the MG cistern and, consequently, ejection [23,139,140] (Figure 4).

OXT plays an essential role in milk ejection, and mice without OXT could not breastfeed their pups [141,142]. Likewise, it has been reported that, during lactation, the activation of the OXT receptor in the MG is observed [143,144]. Intracellular calcium stores are released to generate auto excitation during the next release of OXT and the mobilization of this hormone from dense core vesicles, favoring its more efficient release during the rest of lactation [143,145].

Although OXT is the main hormone associated with milk ejection, hormones such as PRL, cortisol, and the growth hormone, among others, also participate in this process [146,147]. In mammals such as sheep, mice, and cattle, blood OXT concentrations have been associated with a decrease in stress hormones due to the decrease of the action of the HPA axis, reducing the concentration of plasma cortisol plasma [140,148,149]. Wagner et al. [150] evaluated blood cortisol concentration in lactating and non-lactating female *Bos taurus* with or without OXT administration. In non-lactating animals, cortisol concentrations were higher (*p* = 0.02), but the authors concluded that OXT administration alone could not reduce cortisol levels. In contrast, Cook [151] indicated that, in lactating and non-lactating sheep, the variation in blood cortisol could be caused by environmental stress, with or without handling. This factor is also associated with PRL and OXT in the posterior pituitary and the PVN, where there was a lower blood cortisol concentration in non-lactating animals before the same stimuli for both treatments.

For species whose zootechnical purpose is milk production, milk ejection and flow begin with a pronounced increase in plasmatic OXT before milking, highlighting the large reservoir available for milk storage in the udder cistern [152]. Studies in mares have reported that milk ejection occurs 30.8 ± 0.97 s after the suckling foal applies stimuli to the udder, with a peak plasma OXT of 15.8 pmol/L during the 30 to 45 s after feeding initiation of milk letdown [152]. Moreover, it has been described that OXT participates during lactation but also influences maternal behavior during this stage. These effects were described by Valros et al. [101] in 21 Yorkshire lactating sows on the 13th day postpartum during three nursings. The authors investigated the relationship between maternal characteristics and OXT, PRL, and somatostatin levels, finding that udder massage, sow–piglet nasal contacts, and non-stressful nursing periods increase OXT and favor positive behaviors of the mother towards her piglets. Therefore, OXT is a hormone associated with the efficiency of dairy farms, particularly in cattle, the utilization of individual female reserves and increased offspring growth.

An essential element during lactation is reducing stressors to achieve adequate milk ejection [8,68,70]. These characteristics depend on the species. In the case of water buffalo, it has been described that avoiding screams, strange sounds, and changes in the milking process and place prevent the activation of the hypothalamic–pituitary axis and the sympathetic–adreno–medullary axis [153,154]. Since both endocrine axes cause the release of catecholamines and glucocorticoids (e.g., adrenaline and cortisol, respectively), this inhibits the circulation and action of OXT in the myoepithelium of MG, where the portion of milk in the cistern is 5%. Moreover, the alveolar portion represents 95%, making OXT even more relevant for evacuating the alveolar portion [23,155,156]. Routines such as maintaining contact with the calf before and during milking facilitate continuous milk ejection [157].

In addition to providing a comfortable environment, an adequate lactation diet during gestation and after parturition can also activate OXT neurons, as reported in Holstein cows consuming 0.67 kg of dry matter fiber per day [158]. At the same time, parity is associated with higher OXT peak values in multiparous females [159,160,161]. Likewise, allowing the adequate stimulation of MG by the young or establishing other physical tools in dairy farms, such as mechanical brushes to improve well-being, increased affiliative social behaviors and milk yield (1.52 kg) in 72 Holstein (32%) and Swedish Red (68%) cows [158]. The use of this technology in conjunction with vibratory stimulation on MG reported higher plasmatic OXT levels (*p* < 0.05) when comparing Holstein Friesian cows without/with brushing and without/with stimulation by vibration (with 4.96 ± 0.6 pg/mL vs. 25.56 ± 7.3 pg/mL, respectively). However, the amount of residual milk was not affected by treatment and stayed within a normal range (14.9–15.8%) [162]. Exposure to auditory stimuli such as classical, lullaby, or meditation music has also been shown to have physiological benefits in dairy cattle, improving heart and respiratory rates [163].

On the other hand, exogenous administration of OXT in animal production is a common therapeutic practice to treat uterine inertia, placental retention and incomplete abortion, prevent bleeding after parturition, facilitate milk ejection and reduce milking times [5,164,165,166]. However, there is controversy surrounding its use, since it directly influences the productive parameters and the total kg per lactation curve and is an invasive methodology that might compromise animal welfare [167]. The type and frequency of milking and breed also modify these values, as well as the gender of the offspring, as reported in female lambs that increased milk ejection, compared to newborn males [168].

Some of the reported adverse effects are changes in the physicochemical analysis of the milk and impacts on the reproductive health of females. For example, the effect of long-term administration of OXT on pregnancy rates of 23 Nili Ravi buffaloes was studied by Murtaza et al. [169]. The animals received two doses of OXT twice daily (10 and 30 IU) for 154 days post-calving. In general, in animals receiving the hormone, pregnancy rates decreased (*p* < 0.05), more artificial insemination (AI) per conception was required, and the presentation of fetal losses increased in comparison to animals not receiving OXT [169]. A similar finding was reported in 430 cows (Holstein Friesian and Swedish-Red) by Gümen et al. [170]. After AI and administration of OXT intramuscularly (50 IU), conception rates were lower (*p* = 0.02) than those registered in control groups (between 29.3–35.5% vs. 44.2–57.1%, respectively). In the case of ewes, after cervical insemination in 300 animals, a practice where OXT is used to dilate the cervix, lambing rates decreased from 42–69% to 10–52%. However, in these animals, litter size was not affected by OXT treatment [171]. Contrarily, the pregnancy rate in lactating dairy cows increased after OXT administration. Nonetheless, since the sample size was small (17 animals) [172], it is important to mention that the reported differences in these studies can be attributed to different dosages, routes of administration, period of treatment, time to pregnancy diagnosis, and even species.

Concerning the productive affectations in Sahiwal cattle, when groups that did not receive exogenous OXT (MG) and those with 20 IU were compared, a reduction in the percentages of fat (4.49 ± 0.11 G1 vs. 4.49 ± 0.11 G2), lactose (4.84 ± 0.04 G1 vs. 4.37 ± 0.22 G2), protein (3.75 ± 0.08 G1 vs. 3.49 ± 0.15), total solids (13.84 ± 0.20 G1 vs. 13.84 ± 0.20 G2) and non-fat solids (9.35 ± 0.11 G1 vs. 8.73 ± 0.34 G2) was found in the OXT group. In addition, it was found that the application of OXT considerably influenced the mineral profile in milk, causing negative variations in the manufacture of dairy products and by-products [165]. Morgan et al. [173] reported in 30 primiparous ewes that administration of OXT at three doses (1, 5, and 10 IU) did not affect milk yield. However, differences in fat percentages were found, having the lowest values at milk-out (0 h) (7.01%) in animals receiving 1 IU, rather than higher doses of OXT (7.81%). In contrast, sows treated with 75 IU of intramuscular OXT improved milk composition with higher numbers of solids (*p* < 0.05), protein (*p* < 0.01), energy (*p* < 0.05), and even higher IgA concentrations [174].

According to these results, the effect of OXT on milk composition, milk yield, and reproductive health of females depends on the lactation phase, parity, species, pregnancy periods, dosages, and administration routes. Therefore, these elements must be considered when assessing the advantages and disadvantages of OXT.

Furthermore, if circulating OXT levels are low because the mother is in threatening or challenging environments, insufficient milk ejection results, causing inconsistencies and resulting in incomplete emptying of alveolar and cisternal fractions of MG, harming the productive levels, the health status of the MG (e.g., with the onset of mastitis), and the survival of the offspring [147,151]. For this reason, auditory, visual, and tactile tools, and even the administration of exogenous OXT, have been implemented to avoid said deficiencies. However, continuous analysis of these practices is necessary to know their scope and the positive and negative impact they could have on the lactation process and thus encourage its continued use or eradication.

## 6. Future Directions

The possible perspectives of the study concerning OXT could be aimed at assessing the synthesis sites of OXT in tissues other than the myometrium. For example, this hormone can be synthesized in vascular tissue, where it may have a local effect, allowing regulation of the vasomotor response. However, it is still unclear if this could affect the myometrium or if it can alter the behavioral response in animals.

The investigations of OXT in different anatomical regions and compounds are another research field. Recent studies in rabbits have shown that OXT antagonist administration does not impair maternal behavior [175]; however, studies by Keverne and Kendrick [99] have reported that OXT’s effect on maternal neuroendocrine regulation is affected by the administration of opioid receptor blockers, limiting OXT release, a relevant issue when using analgesic treatment or other therapeutic protocols during parturition [27]. Moreover, nursing and maternal traits are highly motivated by parity, species, experience, and intrapartum events such as dystocia [87]. Therefore, further research must address the hormonal response that elicits maternal behavior as a complex phenomenon, not solely dependent on OXT or other neuropeptides.

Regarding the exogenous management of OXT, although it is used to reduce expulsion times, improve milk yield and ejection, and reduce newborn mortality [47,48,49], it is a controversial practice because of the potential adverse effects on dairy animals [5], such as, e.g., intervals between calving and retracements in ovulation [169], and productive effects (impact on milk composition) [23]. On the other hand, it is also necessary to investigate positive stimuli that have been promising, such as the use of brushes, massage [176], music [163], synthetic analogues of maternal appeasing pheromones [177], and the acquisition of a pre-calving milking routine [153,154] to improve dairy cow performance and/or milk parameters. A better understanding of the properties of oxytocinergic cells and the physiology of OXT secretion could also help improve milking machines, for instance, by better synchronizing them with the natural bursts of OXT secretion from the PVN OXT neurons to promote an appropriate hormone release and an efficient/complete milk release, especially in small ruminants [178].

The influence of OXT in species where allomaternal care is present could help to understand the importance of this hormone in both domestic animal behavior and physiology.

## 7. Conclusions

Parturition in mammals is an event coordinated mainly by OXT and its positive feedback loop to maintain constant and synchronous contractions. Although OXT is essential for the onset of parturition, other hormones such as E2, P4, and PG influence the onset of parturition and myometrial contractions.

Immediately after parturition, maternal attachment and behaviors aimed at caring, nursing, and protecting the newborn are highly influenced by increases in serum, milk, and salivary OXT. In several domestic species, low levels of OXT are associated with impaired maternal fitness and delayed or deficient dam–young bonding. The neuroendocrinal management of maternal traits is a relevant topic because it is not only associated with behaviors towards the newborn but also with altruistic care for non-filial offspring, known as allo-nursing, and because neonatal survival is strictly dependent on maternal behavior.

Likewise, the role of OXT in lactation and milk ejection involves the recognition of sensorial stimulus that promotes OXT release and its action on myoepithelial cells located in the alveolus. Due to its importance, exogenous OXT is common in dairy production systems. However, before continuing with its administration, the potential adverse effects must be considered. Therefore, OXT is one of the main hormones influencing physiological and behavioral traits in parturient domestic animals.

## Data Availability

Not applicable.
